# Manganese Superoxide Dismutase (*SOD2*) Polymorphisms, Plasma Advanced Oxidation Protein Products (AOPP) Concentration and Risk of Kidney Complications in Subjects with Type 1 Diabetes

**DOI:** 10.1371/journal.pone.0096916

**Published:** 2014-05-12

**Authors:** Kamel Mohammedi, Naïma Bellili-Muñoz, Fathi Driss, Ronan Roussel, Nathalie Seta, Frédéric Fumeron, Samy Hadjadj, Michel Marre, Gilberto Velho

**Affiliations:** 1 Research Unit 1138, INSERM, Paris, France; 2 Department of Diabetology, Endocrinology and Nutrition, Assistance Publique Hôpitaux de Paris - Bichat Hospital, Paris, France; 3 Research Unit 773, INSERM, Paris, France; 4 Department of Biochemistry, Assistance Publique Hôpitaux de Paris - Bichat Hospital, Paris, France; 5 UFR de Médecine, Univ Paris Diderot, Sorbonne Paris Cité, Paris, France; 6 UFR de Pharmacie, Univ Paris Diderot, Sorbonne Paris Cité, Paris, France; 7 Department of Endocrinology and Diabetology, Centre Hospitalier Universitaire de Poitiers, Poitiers, France; 8 Research Unit 1082, INSERM, Poitiers, France; 9 Centre d'Investigation Clinique (CIC) 0802, INSERM, Poitiers, France; 10 UFR de Médecine et Pharmacie, Université de Poitiers, Poitiers, France; National Centre for Scientific Research “Demokritos”, Greece

## Abstract

**Aims:**

Oxidative stress is involved in the pathophysiology of diabetic nephropathy. Manganese superoxide dismutase (SOD2) catalyses the dismutation of superoxide, regulates the metabolism of reactive oxygen species in the mitochondria and is highly expressed in the kidney. Plasma concentration of advanced oxidation protein products (AOPP), a marker of oxidative stress, was found to be increased in patients with kidney disease. We investigated associations of *SOD2* allelic variations, plasma SOD activity and AOPP concentration with diabetic nephropathy in type 1 diabetic subjects.

**Methods:**

Eight SNPs in the *SOD2* region were analysed in 1285 Caucasian subjects with type 1 diabetes from the SURGENE prospective study (n = 340; 10-year follow-up), GENESIS (n = 501) and GENEDIAB (n = 444) cross-sectional studies. Baseline plasma concentration of AOPP and SOD activity were measured in GENEDIAB participants. Hazard ratio (HR) and odds ratio (OR) were determined for incidence and prevalence of nephropathy. Analyses were adjusted or stratified by retinopathy stages.

**Results:**

In the SURGENE cohort, the T-allele of rs4880 (V16A) was associated with the incidence of renal events (new cases, or the progression to a more severe stage of nephropathy; HR 1.99, 95% CI 1.24–3.12, p = 0.004) and with the decline in estimated glomerular filtration rate (eGFR) during follow-up. Similar associations were observed for rs2758329 and rs8031. Associations were replicated in GENESIS/GENEDIAB cohorts, in the subset of participants without proliferative retinopathy, and were confirmed by haplotype analyses. Risk allele and haplotype were also associated with higher plasma AOPP concentration and lower SOD activity.

**Conclusions:**

*SOD2* allelic variations were associated with the incidence and the progression of diabetic nephropathy, with a faster decline in eGFR and with plasma AOPP concentration and SOD activity in subjects with type 1 diabetes. These results are consistent with a role for *SOD2* in the protection against oxidative stress and kidney disease in type 1 diabetes.

## Introduction

Diabetic nephropathy is a major cause of end-stage renal disease [1], and is associated with increased cardiovascular and all-cause mortality, accounting for most of the reduced life expectancy of patients with type 1 diabetes [2]. Diabetic nephropathy is strongly dependent on poor blood glucose control [3]. However, it is now clear that genetic factors modulate the individual susceptibility to diabetic nephropathy [4]–[8].

Reactive oxygen species (ROS) are produced in all cells as part of the normal cellular metabolism [9]. The mitochondrial respiratory chain produces large amounts of superoxide radicals and is the major source of intracellular ROS [10]. Oxidative stress occurs when production of ROS exceeds local antioxidant capacity. In this situation, there is increased oxidation of proteins, lipids, carbohydrates and DNA, that can result in tissue and organ damage. Hyperglycaemia increases the production of ROS and causes oxidative stress [11]. Oxidative stress influences multiple pathways implicated in diabetic nephropathy [11], [12]. Advanced oxidation protein products (AOPP) were identified as markers of oxidative stress in patients with kidney disease [13].

Superoxide dismutase (SOD) is a group of enzymes that catalyse the dismutation of superoxide into oxygen and hydrogen peroxide [14], playing an important role in antioxidant mechanism in cells exposed to oxygen. Three isoforms of SOD are expressed in humans, encoded by different genes. SOD1 (Copper-zinc superoxide dismutase, Cu-Zn SOD) is located in the cytoplasm [14] and SOD3 (extracellular superoxide dismutase, EC-SOD) is a major extracellular antioxidant enzyme [15]. Manganese superoxide dismutase (Mn-SOD or SOD2) is located mainly in the inner mitochondrial membrane and is highly active in the human kidney [16]. Previous investigations reported associations of a missense variant in the exon 2 of the *SOD2* gene (rs4880: V16A) with diabetic nephropathy in subjects with type 1 diabetes [17], [18]. In the present study, we investigated associations of a set of *SOD2* tagSNPs, plasma AOPP concentration and SOD activity with diabetic nephropathy in type 1 diabetic subjects.

## Methods

### Participants

We studied three cohorts designed to evaluate the genetic components of diabetic nephropathy. The “Survival Genetic Nephropathy” (SURGENE) was a prospective study conducted in 340 subjects with type 1 diabetes, recruited consecutively over a 3-year period in the outpatient clinic of the University Hospital of Angers, France [19]. At baseline, 60 individuals (17.6%) had a diagnosis of incipient, established or advanced diabetic nephropathy (see below). Mean duration of follow-up was 10±3 years. New cases of incipient nephropathy (persistent microalbuminuria in consecutive biannual assessments) during follow-up were observed in 76 out of the 280 normoalbuminuric subjects at baseline (27.1%). A renal event, defined as new cases of incipient nephropathy or progression to a more severe stage of nephropathy, was observed in 98 (28.8%) participants during the follow-up. At the end of the study 136 individuals (40%) had diagnosis of incipient, established or advanced diabetic nephropathy. The GENESIS France-Belgium Study and the “Génétique de la Néphropathie Diabétique” (GENEDIAB) study were cross-sectional, multi-centre, binational (Belgium and France) studies. GENESIS was family-based, including 662 probands with type 1 diabetes and presenting with diabetic retinopathy [20]. We studied 501 probands for whom DNA samples were available, including 279 individuals (55.7%) with diagnosis of incipient, established or advanced diabetic nephropathy. GENEDIAB included 444 patients with type 1 diabetes and diagnosis of severe diabetic retinopathy [4]. Incipient, established or advanced diabetic nephropathy was present in 310 individuals (69.8%).

### Ethics Statement

This investigation was conducted according to the principles expressed in the Declaration of Helsinki. All participants gave written informed consent and study protocols were approved by the ethics committee of the University Hospital of Angers, France.

### Diabetic nephropathy and retinopathy stages

Stages of diabetic nephropathy were defined as follows: no nephropathy, defined as urinary albumin excretion (UAE) <30 mg/24 h or <20 µg/min or <20 mg/l and plasma creatinine <150 µmol/l in at least two of three consecutive assessments and in the absence of antihypertensive treatment; incipient nephropathy, defined as persistent microalbuminuria (UAE = 30–300 mg/24 h or 20–200 µg/min or 20–200 mg/l) and plasma creatinine <150 µmol/l in at least two of three consecutive assessments; established nephropathy, defined as past or present macroalbuminuria (UAE>300 mg/24 h or >200 µg/min or >200 mg/l) and plasma creatinine <150 µmol/l; and advanced nephropathy, defined as past or present macroalbuminuria and plasma creatinine >150 µmol/l or renal replacement therapy. Estimation of the glomerular filtration rate (eGFR) was computed with the Modification of Diet in Renal Disease (MDRD) formula [21]. Retinopathy was staged according to Kohner's classification [22] as non-proliferative, pre-proliferative or proliferative retinopathy.

### Laboratory procedures

Plasma concentration of AOPP (n = 381) and plasma SOD activity (n = 371) were measured in GENEDIAB participants for whom fasting plasma-EDTA samples were available. Plasma-EDTA samples were collected at baseline and kept frozen at −80°C. AOPP concentration was measured by spectrophotometry using a microplate reader (MR 5000, Dynatech, Paris, France) [13]. SOD activity was measured using a quantitative colorimetric assay kit (EnzyChrom™ ESOD-100, BioAssay Systems, Hayward, CA). Eight SNPs (rs4342445, rs2758329, rs2842980, rs7855, rs8031, rs5746141, rs5746136 and rs4880) in the SOD2 region (chr.6q25.3) were analysed. The SNPs were chosen in HapMap (public release #23) on the basis of giving information on ∼90% of the allelic variation of SNPs with minor allele frequency ≥5% at r2>0.8 in haplotype blocks containing SOD2. Genotypes were determined by competitive allele-specific PCR genotyping system assays (KASP, LGC Genomics, Hoddeston, UK). Genotyping success rate was >95%. Genotyping was repeated in 5% of subjects with 100% concordance. All genotypes were in Hardy-Weinberg equilibrium.

### Statistical analysis

Results are expressed as mean ± SD except where stated otherwise. For all analyses of quantitative parameters, data were log-transformed when the normality of the distribution was rejected by the Shapiro-Wilk W test. Differences between groups were assessed by Pearson's chi-squared test, Fisher's exact test, Wilcoxon/Kruskal-Wallis test, ANOVA and ANCOVA. When ANOVA or ANCOVA were significant, comparisons between pairs were made using the Tukey-Kramer HSD test. Variation of eGFR during the study was computed as the difference between values at the end of follow-up and at baseline, divided by the duration of follow-up, and expressed in ml/min.year^−1^. Associations with diabetic nephropathy were assessed by regression models. Cox proportional hazards survival regression analyses were used to examine the effect of explanatory variables on time-related survival (microalbuminuria-free or renal event-free) rates in prospective analyses. Logistic regression analyses were used for cross-sectional analyses. Hazard ratios (HR) or odds ratios (OR), respectively, with their 95% confidence intervals (CI) were computed in these analyses for the major alleles. To increase power, subjects with established or advanced nephropathy were pooled for genetic analyses. To take into account differences in diabetic retinopathy status in different cohorts resulting from different inclusion criteria, and because of a possible *SOD2* genotype confounding effect on retinopathy [23], [24], all comparisons of diabetic nephropathy by genotype included adjustment or stratification for retinopathy stages. Stratification by retinopathy stage (non-proliferative/pre-proliferative vs proliferative) of genotype effects on nephropathy was performed by nesting the genotype variable within the retinopathy stage variable in the regression analysis. This results in the computation of statistical effects for non-proliferative/pre-proliferative and proliferative retinopathy sub-groups of individuals separately, and adjusted for multiple comparisons due to the stratification by retinopathy stages. Correction for multiple comparisons due to multiple SNP testing took into account the effective number of independent tests (M_eff_) based on the degree of linkage disequilibrium between SNPs [25]. Thus, p≤0.01 was considered significant for genotype-related comparisons, unless stated otherwise. The power to detect associations of the SNPs with the incidence of renal events during follow-up and the prevalence of established/advanced nephropathy at the end of the study in the SURGENE cohort, and with the prevalence of established/advanced nephropathy in the cross-sectional study was 0.56, 0.64 and 0.99, respectively, for odds ratio or hazard ratio ≥1.5 and alpha = 0.01. It was 0.72, 0.84 and 0.99, respectively for alpha = 0.05. The power to detect a 20–25% variation of plasma AOPP concentrations and SOD activity was ∼80%. Statistics were performed with the JMP software (SAS Institute Inc., Cary, NC). Linkage disequilibrium (LD) and haplotype analyses were performed with the THESIAS software v3.1 [26].

## Results

### Prospective SURGENE study: renal events during follow-up by *SOD2* genotype

**Figure 1:**
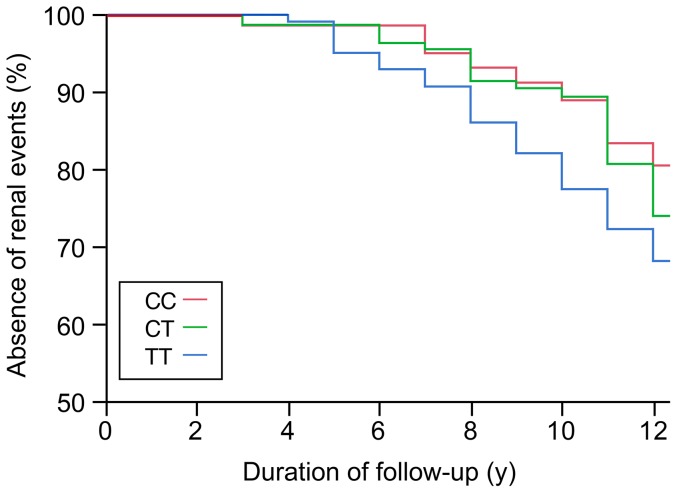
Kaplan-Meier survival (renal event-free) curve during follow-up in the SURGENE cohort by *SOD2* rs4880 genotypes: TT (n = 108), TC (n = 158), CC (n = 74). Y-axis represents the absence of renal events defined as new cases of microalbuminuria or progression to a more severe stage of nephropathy.

The prevalence of incipient, established and advanced diabetic nephropathy at baseline was 10.6%, 5.3% and 1.7%, respectively. Characteristics of participants by stages of nephropathy at baseline were published previously [7]. The incidence of renal events (new cases of microalbuminuria or progression to a more severe stage of nephropathy) during the follow-up was 28.8% (n = 98). Individuals who presented a renal event, as compared to those with stable renal status during follow-up, had at baseline a longer duration of diabetes, higher HbA1c, triglycerides and blood pressure levels, decreased renal function, and were more often taking ACE inhibitors and antihypertensive medication (Table 1). Diabetic retinopathy was more frequent and severe in individuals who presented a renal event. Macrovascular complications (myocardial infarction, stroke and/or peripheral artery disease) were also more frequent in these subjects. The incidence of renal events by genotype was 34.4% (TT), 24.1% (TC) and 27.5% (CC) for rs2758329, 33.7% (TT), 24.6% (TA) and 27.5% (AA) for rs8031, and 34.0% (TT), 25.0% (TC) and 28.4% (CC) for rs4880, suggesting possible recessive effects of the major alleles of these variants. Cox proportional hazards survival regression analyses showed associations of 3 SNPs (rs2758329, rs8031 and rs4880) with the incidence of renal events (Table 2 and Figure 1). These associations remained significant when we considered only the incident cases of microalbuminuria during the follow-up (27.1%): HR 1.56, 95% C.I. 1.13–2.14, p = 0.008 for rs2758329, HR 1.55, 95% C.I. 1.12–2.13, p = 0.009 for rs8031, and HR 1.54, 95% C.I. 1.11–2.11, p = 0.01 for rs4880. At the end of follow-up, the total prevalence of incipient and established/advanced nephropathy was 28.8% and 11.2%, respectively. Four SNPs were associated with the total prevalence of established/advanced nephropathy at the end of follow-up: OR 4.24, 95% CI 1.86–10.68, p = 0.0004 for rs2758329, OR 4.19, 95% CI 1.85–10.54, p = 0.0004 for rs8031, OR 0.30, 95% CI 0.11–0.72, p = 0.009 for rs5746136, and OR 4.01, 95% CI 1.80–9.88, p = 0.0005 for rs4880 OR for the major alleles in a codominant model adjusted for sex, age, duration of diabetes, treatment by ACE inhibitors, and diabetic retinopathy stages).

**Table 1:** SURGENE cohort: Characteristics of participants at baseline by incidence of renal events during the follow-up.

	Incidence of renal events		
	No	Yes	p
N	242	98	
Age (years)	33±13	34±13	0.45
Sex: M/F (%)	46/54	37/63	0.12
Age at diabetes onset (years)	19±10	18±9	0.37
Duration of diabetes (years)	14±11	17±11	0.01
HbA_1c_ (mmol/mol)(%)	77±25 (9.2±2.3)	84±25 (9.8±2.3)	0.02
BMI (kg/m^2^)	22.9±3.1	22.7±3.2	0.62
Systolic blood pressure (mmHg)	124±14	133±18	<0.0001
Diastolic blood pressure (mmHg)	72±10	75±12	<0.0001
Plasma creatinine (µmol/l)	82±35	108±110	0.0007
eGFR (ml/min)	98±23	91±26	0.009
Urinary albumin excretion (mg/l)*	7 (9)	10 (13)	0.001
Total Cholesterol (mmol/l)	5.43±1.40	5.38±1.19	0.89
LDL Cholesterol (mmol/l)	3.47±1.34	3.23±1.10	0.24
HDL Cholesterol (mmol/l)	1.53±0.52	1.47±0.44	0.51
Triglycerides (mmol/l)*	0.85 (0.49)	1.13 (0.70)	0.0005
Hypolipaemic Treatment (%)	2.8	2.3	0.81
ACE Inhibitor treatment (%)	5.8	15.3	0.009
Antihypertensive treatment (%)	8.3	22.4	0.0008
Diabetic retinopathy stages (%)	67/14/12/7	48/18/15/19	0.003
Macrovascular complications (%)	1.2	5.1	0.05

Results expressed as means ± SD or *median and interquartile range. Statistics of quantitative parameters are ANOVA performed with log-transformed data or *Wilcoxon (rank sums) test. p<0.05 is significant. Diabetic retinopathy stages: Absence, Non-Proliferative, Pre-Proliferative, Proliferative. Macrovascular complications include all cases of myocardial infarction, stroke and/or peripheral artery disease.

**Table 2:** SURGENE cohort: Genotype frequencies of *SOD2* polymorphisms by incidence of renal events during follow-up.

	Renal events			
SNP	No (n = 242)	Yes (n = 98)	HR (95% C.I.)	p
rs4342445				
GG	0.584	0.622	1.08	0.72
GA	0.353	0.347	(0.70–1.70)	
AA	0.063	0.031		
MAF	0.240	0.204		
rs2758329				
TT	0.283	0.381	1.94	0.006
TC	0.493	0.392	(1.22–3.04)	
CC	0.224	0.227		
MAF	0.471	0.423		
rs2842980				
AA	0.590	0.532	0.79	0.28
AT	0.360	0.372	(0.51–1.22)	
TT	0.050	0.096		
MAF	0.230	0.282		
rs7855				
AA	0.874	0.887	1.03	0.93
AG	0.126	0.113	(0.51–1.89)	
GG	0	0		
MAF	0.063	0.057		
rs8031				
TT	0.293	0.381	1.90	0.007
TA	0.482	0.392	(1.19–2.98)	
AA	0.225	0.227		
MAF	0.466	0.423		
rs5746141				
GG	0.929	0.857	0.66	0.21
GA	0.067	0.143	(0.36–1.30)	
AA	0.004	0		
MAF	0.038	0.071		
rs5746136				
GG	0.528	0.479	1.10	0.61
GA	0.367	0.469	(0.62–1.42)	
AA	0.105	0.052		
MAF	0.289	0.286		
rs4880				
TT	0.295	0.381	1.99	0.004
TC	0.484	0.392	(1.24–3.12)	
CC	0.221	0.227		
MAF	0.463	0.423		

SNPs are sorted in 5′ to 3′ order. Hazards ratio for the major allele in a recessive model (MM vs Xm) determined in Cox proportional hazards survival regressive model, adjusted for sex, age, duration of diabetes, treatment by ACE inhibitors and retinopathy stages. Retinopathy was coded as an ordinal polytomic covariate: absent (1), non-proliferative (2), pre-proliferative (3), or proliferative retinopathy (4). MAF: minor allele frequency. p≤0.01 is significant.

### Prospective SURGENE study: estimated renal function by *SOD2* genotype

**Figure 2:**
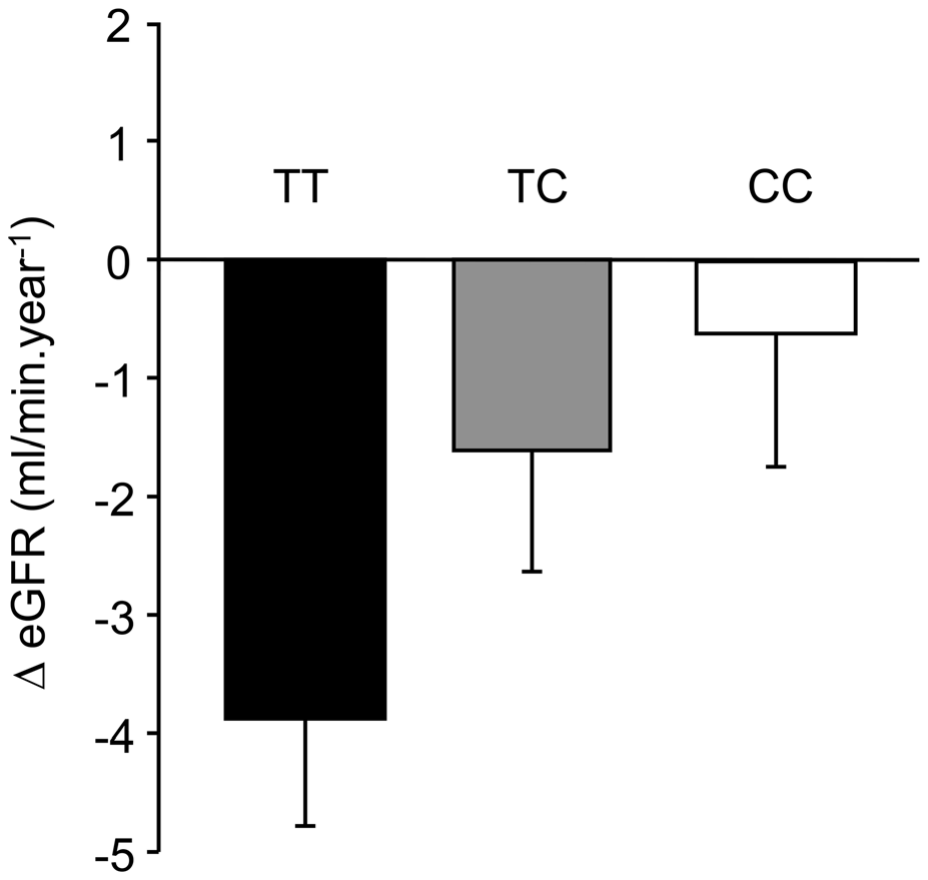
Variation of eGFR (ml/min.year^−1^) during follow-up in the SURGENE cohort by *SOD2* rs4880 genotypes: TT (n = 108), TC (n = 158), CC (n = 74). Results expressed as means ± SEM.

No genotype-related differences in eGFRs were observed at baseline (data not shown). The variation of eGFR during follow-up by rs4880 genotype is shown in figure 2. It was −3.96±0.89 (TT), −1.57±1.05 (TC) and −0.64±1.12 ml/min.year^−1^ (CC) (mean ± SEM, ANCOVA p = 0.003, adjusted for sex, age, duration of diabetes, and treatment by ACE inhibitors). The risk alleles of rs8031 and rs2758329 were also associated with a more severe decrease in eGFR during follow-up (data not shown).

### Cross-sectional GENESIS and GENEDIAB studies

Characteristics of participants with or without nephropathy from GENESIS and GENEDIAB studies were published previously [7]. Briefly, in both cohorts, individuals with established or advanced nephropathy as compared to those with normal UAE had higher blood pressure levels, lower eGFR, had more frequently a past or present history of smoking, and were more likely to be taking ACE inhibitors and antihypertensive medication. Participants from GENEDIAB with established or advanced nephropathy had higher levels of total cholesterol and triglycerides as compared to levels in participants with normoalbuminuria. Proliferative retinopathy was present in 56% and 82% of the participants from GENESIS and GENEDIAB cohorts, respectively, reflecting different inclusion criteria. To increase statistical power, genetic analyses were performed in pooled cohorts with stratification by retinopathy stage (non-proliferative/pre-proliferative vs proliferative) and appropriate adjustments for covariates to take into account cohort differences. Clinical characteristics of subjects by retinopathy status are shown in Table S1. Subjects with proliferative retinopathy as compared to those with non-proliferative or pre-proliferative retinopathy, were older, had a younger age of diagnosis and a longer duration of diabetes, higher blood pressure levels, lower BMI and eGFR, and were more likely to be taking ACE inhibitors and antihypertensive medication. Macrovascular diseases (myocardial infarction, stroke, peripheral artery disease) were more frequent, and diabetic nephropathy was more frequent and more severe in individuals with proliferative retinopathy. Genotype frequencies by stages of nephropathy in the subset of subjects with non-proliferative/pre-proliferative retinopathy are shown in Table 3. We observed associations of rs2758329, rs8031 and rs4880 with established/advanced nephropathy in a codominant model. These results are in keeping with genotype-related prevalence of established/advanced nephropathy: 25.2% (TT), 25.2% (TC) and 14.3% (CC) for rs2758329, 25.2% (TT), 23.9% (TA) and 14.7% (AA) for rs8031, and 27.5% (TT), 25.9% (TC) and 13.3% (CC) for rs4880. We have also observed nominal associations of rs4342445 (p = 0.03) and rs5746136 (p = 0.03) with established/advanced nephropathy. We have not observed any association of the SNPs with diabetic nephropathy in the subset of participants with proliferative retinopathy (data not shown).

**Table 3:** GENESIS and GENEDIAB pooled studies – subset of participants with non-proliferative or pre-proliferative retinopathy: genotype frequencies of *SOD2* polymorphisms by stages of diabetic nephropathy.

SNP	Absence of nephropathy (n = 187)	Incipient nephropathy (n = 95)	Established or advanced nephropathy (n = 84)	OR (95% C.I.) for incipient nephropathy	p	OR (95% C.I.) for established or advanced nephropathy	p
rs4342445							
GG	0.644	0.567	0.468	0.88	0.60	0.54	0.03
GA	0.294	0.400	0.456	(0.53–1.47)		(0.31–0.94)	
AA	0.061	0.033	0.076				
MAF	0.208	0.233	0.304				
rs2758329							
TT	0.309	0.269	0.338	0.98	0.64	2.08	0.005
TC	0.438	0.505	0.525	(0.68–1.38)		(1.26–3.53)	
CC	0.253	0.226	0.137				
MAF	0.472	0.478	0.400				
rs2842980							
AA	0.629	0.685	0.603	1.06	0.62	1.10	0.89
AT	0.309	0.283	0.384	(0.62–1.85)		(0.70–1.76)	
TT	0.062	0.032	0.013				
MAF	0.216	0.174	0.205				
rs7855							
AA	0.881	0.914	0.912	2.05	0.17	1.59	0.35
AG	0.119	0.086	0.088	(0.73–6.46)		(0.65–4.35)	
GG	0	0	0				
MAF	0.060	0.043	0.044				
rs8031							
TT	0.313	0.272	0.351	0.88	0.67	1.99	0.009
TA	0.437	0.511	0.506	(0.25–1.28)		(1.20–3.36)	
AA	0.250	0.217	0.143				
MAF	0.469	0.473	0.396				
rs5746141							
GG	0.873	0.880	0.899	1.28	0.66	1.33	0.49
GA	0.121	0.109	0.101	(0.44–3.95)		(0.61–3.25)	
AA	0.006	0.011	0				
MAF	0.066	0.065	0.051				
rs5746136							
GG	0.528	0.402	0.385	0.77	0.26	0.54	0.03
GA	0.376	0.533	0.500	(0.47–1.27)		(0.31–0.94)	
AA	0.096	0.065	0.115				
MAF	0.284	0.332	0.365				
rs4880							
TT	0.284	0.264	0.337	1.13	0.60	2.16	0.005
TC	0.449	0.516	0.530	(0.72–1.80)		(1.28–3.72)	
CC	0.267	0.220	0.133				
MAF	0.491	0.478	0.398				

SNPs are sorted in 5′ to 3′ order. OR (odds ratio) for the major allele in a codominant model determined in logistic regression analyses adjusted for sex, age, duration of diabetes, cohort membership and treatment by ACE inhibitors. MAF: minor allele frequency. p≤0.01 is significant.

### Haplotype analyses

LD between SNPs was computed and haplotype frequencies of the variants associated with diabetic nephropathy (rs2758329, rs8031, rs5746136 and rs4880) were determined in SURGENE and in pooled GENESIS/GENEDIAB cohorts. Logistic regression analyses were performed with the nephropathy stage as the dependent variable and the haplotypes as the independent covariate (Table 4). We observed in the SURGENE cohort an association of the TTAT haplotype, composed of the risk alleles of the 4 SNPs, with established/advanced nephropathy at the end of follow-up (baseline plus incident cases). Haplotype analyses confirmed the association of the TTAT haplotype with established/advanced nephropathy in the subset of patients from GENESIS/GENEDIAB cohorts with non-proliferative/pre-proliferative retinopathy (Table 4), but not in subjects with proliferative retinopathy (data not shown).

**Table 4:** Haplotype frequencies by stages of diabetic nephropathy in SURGENE and pooled GENESIS/GENEDIAB studies.

Haplotypes	Absence of nephropathy	Incipient nephropathy	Established or advanced nephropathy	OR (95% C.I.) for incipient nephropathy	p	OR (95% C.I.) for established or advanced nephropathy	p
SURGENE	(n = 204)	(n = 98)	(n = 38)				
CAGC	0.458	0.461	0.355	1		1	
TTAT	0.286	0.269	0.366	1.10 (0.71–1.71)	0.66	3.06 (1.32–7.09)	0.009
TTGT	0.237	0.258	0.263	1.30 (0.83–2.04)	0.26	2.62 (0.99–6.98)	0.05
TTGC	0.013	0.006	0.016	-	-	-	-
CAGT	0.006	0.006	0.000	-	-	-	-
GENESIS/GENEDIAB	(n = 187)	(n = 95)	(n = 84)				
CAGC	0.472	0.473	0.394	1		1	
TTAT	0.285	0.328	0.368	1.29 (0.83–1.98)	0.26	1.90 (1.17–3.09)	0.009
TTGT	0.217	0.171	0.226	0.87 (0.54–1.39)	0.55	1.45 (0.85–2.48)	0.17
TTGC	0.023	0.022	0.006	-	-	-	-
CAGT	0.003	0.006	0.006	-	-	-	-

Haplotypes represent the alleles of rs2758329, rs8031, rs5746136 and rs4880, respectively. Odds ratios for diabetic nephropathy stages for each haplotype as compared to the odds for the most frequent haplotype (CAGC) considered to be 1. SURGENE data represents prevalence of diabetic nephropathy at the end of follow-up (baseline plus incident cases); analyses adjusted for sex and age. GENESIS/GENEDIAB data represents the subset of participants with non-proliferative or pre-proliferative retinopathy; analyses adjusted for sex, age and cohort membership. p≤0.05 is significant.

### GENEDIAB cohort: Plasma AOPP concentration, plasma SOD activity and diabetic nephropathy

Plasma AOPP concentration was higher in woman than in men (71±3 vs 63±2 µmol/l, mean ± SEM, p = 0.009), and lower in subjects treated by ACE inhibitors (44±2 vs 86±2 µmol/l, p<0.0001) or antihypertensive drugs (56±2 vs 79±2 µmol/l, p<0.0001) than in subjects not receiving these medications. It was positively correlated with levels of total cholesterol (r^2^ = 0.05, p<0.0001) and triglycerides (r^2^ = 0.06, p = 0.01). In a stepwise regression analysis including as independent covariates sex, age, duration of diabetes, total cholesterol, triglycerides and HbA1c levels, diabetic retinopathy status and use of ACE inhibitors and antihypertensive drugs, only use of ACE inhibitors remained inversely correlated (r^2^ = 0.38, p<0.0001) and HbA1c levels positively correlated (r^2^ = 0.01, p = 0.03) with AOPP concentration (data not shown). Plasma AOPP concentration was lower in participants without nephropathy (60±3 µmol/l, n = 131), intermediate in participants with incipient nephropathy (64±4 µmol/l, n = 83) and higher in participants with established/advanced nephropathy (70±3 µmol/l, n = 167, p = 0.03, mean ± SEM, ANCOVA adjusted for sex, age, HbA1c levels and use of ACE inhibitors).

Plasma SOD activity was higher in subjects treated by ACE inhibitors (3.31±0.14 vs 2.24±0.11 U/ml, mean ± SEM, p<0.0001) or antihypertensive drugs (2.97±0.12 vs 2.27±0.13 U/ml, p<0.0001) than in subjects not receiving these medications. SOD activity was positively correlated with age (r^2^ = 0.01, p = 0.04) and inversely correlated with plasma AOPP concentration (r^2^ = 0.04, p<0.0001). In a stepwise regression analysis only use of ACE inhibitors remained inversely correlated with SOD activity (r^2^ = 0.13, p<0.0001) and no independent association or correlation was observed with age, duration of diabetes, HbA1c levels or diabetic retinopathy status (data not shown). Plasma SOD activity was not significantly different by diabetic nephropathy status: 2.95±0.19 (n = 130) vs 2.98±0.21 (n = 81) vs 3.01±0.17 (n = 160) mU/l for subjects without nephropathy, or with incipient or established/advanced nephropathy, respectively (p = 0.97, mean ± SEM, ANCOVA adjusted for sex, age and use of ACE inhibitors).

### GENEDIAB cohort: Plasma AOPP concentration, plasma SOD activity and *SOD2* genotypes and haplotypes

Plasma AOPP concentration by rs4880 genotype was 74±3 (TT), 66±3 (TC) and 57±3 µmol/l (CC), p = 0.0001 (mean ± SEM, ANCOVA adjusted for sex, age and use of ACE inhibitors). The risk alleles of rs2758329, rs8031 and rs5746136 were also associated with significantly higher plasma AOPP concentration (data not shown). The haplotype composed of the risk alleles of rs2758329, rs8031, rs5746136 and rs4880 (TTAT) was associated with significantly higher plasma AOPP concentration than the haplotype composed of the complementary alleles (CAGC): 38 (95% C.I. 34–43) µmol/l per haplotype vs 28 (95% C.I. 25–31) µmol/L per haplotype, p = 0.001 (adjusted for sex, age and use of ACE inhibitors).

No genotype-related differences in plasma SOD activity were observed in the whole set of participants (data not shown). However, in the subset of subjects with established/advanced nephropathy, the risk genotype of rs4880 was associated with significantly lower plasma SOD activity: 2.38±0.26 (TT) vs 3.04±0.15 U/ml (CT/CC), p = 0.008 (ANCOVA, adjusted for sex, age and use of ACE inhibitors). Similar results were observed for the risk genotypes of rs2758329 and rs8031 (data not shown). The haplotype composed of the risk alleles of rs2758329, rs8031, rs5746136 and rs4880 (TTAT) was associated with significantly lower plasma SOD activity than the haplotype composed of the complementary alleles (CAGC): 0.89 (95% C.I. 0.71–1.07) U/ml per haplotype vs 1.18 (95% C.I. 0.96–1.39) U/ml per haplotype, p = 0.05 (adjusted for sex, age and use of ACE inhibitors).

## Discussion

We observed associations of *SOD2* gene variants with diabetic nephropathy in cohorts of subjects with type 1 diabetes. In the prospective SURGENE study, rs2758329, rs8031 and rs4880 were associated with incidences of incipient nephropathy and renal events, and with the decline of eGFR during the follow-up. The same variants were associated with established/advanced nephropathy in participants with non-proliferative/pre-proliferative retinopathy from GENESIS and GENEDIAB cross-sectional studies. These associations were confirmed by haplotype analyses. We have also observed associations of the risk allele and the risk haplotype of these variants with higher plasma AOPP concentration and lower SOD activity in GENEDIAB.

No association of *SOD2* variants with diabetic nephropathy was observed in the subset of participants with proliferative retinopathy in the cross-sectional study. As the number of subjects and thus the statistical power of the study were higher in subjects with proliferative retinopathy, it seems plausible that the different results observed in the two groups of subjects have a biological rather than a purely statistical basis. The reasons underlying the contrasting results observed in the two groups of subjects could include interactions of genotype with diabetic retinopathy and/or differences in the renal phenotype of subjects with different severity of diabetic retinopathy. Contrasting results were observed in a few small studies regarding associations of rs4880 with diabetic retinopathy [23], [24], but a possible confounding effect of diabetic nephropathy was not taken into account in those studies. In the present investigation, we observed no association of *SOD2* variants with retinopathy (data not shown). On the other hand, subjects from GENESIS and GENEDIAB with proliferative retinopathy were more severely ill as compared to those with non-proliferative/pre-proliferative retinopathy. Not only diabetic nephropathy but also arterial hypertension and macrovascular complications were more frequent and/or more severe in individuals with proliferative retinopathy. The prevalence of established/advanced nephropathy was more than twice as high in subjects with proliferative retinopathy as in subjects with non-proliferative/pre-proliferative retinopathy (51% vs 23%). It is tempting to speculate that a more complex and heterogeneous pathophysiology of the kidney disease in subjects with proliferative retinopathy might mask the allelic effects of SOD2. Moreover, given the severity of micro- and macrovascular complications in subjects with proliferative retinopathy, survival bias might have also blunted these allelic effects. Much larger cohorts with detailed intermediate phenotypes would be required to sort out the interactions of microvascular and macrovascular components in the allelic associations with kidney disease.

The main strengths of our study are the detailed phenotype assessment of renal function during the 10-year follow-up of the SURGENE cohort, the replication of our findings in independent cohorts, the assessment of possible confounding effects of diabetic retinopathy, and the genotyping of tagSNPs covering all haplotype blocks containing *SOD2*. Moreover, we also investigated associations of diabetic nephropathy and *SOD2* genotypes with two markers of redox status, AOPP and SOD activity. However, our study has limitations, notably in issues related to the measurement of these markers. Plasma AOPP and SOD activity were assayed in plasma-EDTA samples collected at baseline and kept frozen for ∼20 years, and measurements were performed only in a subset of GENEDIAB participants, from whom plasma samples were available. Moreover, it is noteworthy that plasma SOD activity reflects the activity of all SOD isoforms with Mn-SOD accounting for only ∼10% of total plasma SOD activity [15]. These issues might have affected, at least in part, comparisons of AOPP and SOD activity between groups. Another limitation of the study is the relatively small sample size of our cohorts and small number of renal events in the prospective study. The statistical power was adequate (0.56–0.72%) to detect SNP effects with HR ≥1.5, but might have been insufficient to detect effects of smaller magnitude. On the other hand, it is unlikely that all our findings only reflect type 1 error (false positive results) due to population stratification. Allelic association in the SURGENE cohort was observed with the incidence of microalbuminuria and renal events during follow-up, but also with the evolution of eGFR, an independent trait, during the study. A type 1 error due to population stratification is less likely to occur in a prospective study, much less prone to selection bias, than in case control studies. Moreover, our results corroborate previous findings in the literature [17], [18], [27]. Möllsten and co-workers observed association of the T-allele of rs4880 with increased risk of incipient and overall diabetic nephropathy in Finish and Swedish cross-sectional studies and in a Danish prospective study of subjects with type 1 diabetes [17], [18].

The molecular mechanisms beyond the associations of *SOD2* variants with diabetic nephropathy are not fully elucidated. Rs4880 is located in exon 2 of *SOD2* and results in the replacement of the valine in codon 16 for an alanine (c.T47C, p.V16A). In vitro studies showed the valine residue to be associated with less efficient transport of Mn-SOD into the mitochondrial matrix [28], an effect that could compromise its antioxidant properties. However, contrasting results were observed for the impact of genotype on Mn-SOD activity [29], [30]. In our study, the valine risk allele was associated with higher plasma concentration of AOPP and with lower SOD activity. The other SNPs that we have studied are located outside coding or known regulatory regions. However, the pattern of linkage disequilibrium between rs4880 and the other SNPs in our cohorts suggests that the associations we have observed for rs2758329 and rs8031 with nephropathy could be accounted for by the association of rs4880 with the trait. Linkage disequilibrium (expressed as R^2^) of these variants with rs4880 was higher than 0.90 in both cohorts, while it was 0.06–0.25 for the SNPs not associated with nephropathy. Less clear-cut results were observed for rs5746136 (R^2^: 0.36).

Oxidative stress plays a key role in the development of diabetic nephropathy [12]. Overproduction of ROS induced by hyperglycaemia is an early molecular mechanism in the pathogenesis of diabetic kidney complications [11], [31]. High glucose concentrations increase the formation of ROS and leads to oxidative stress by several molecular mechanisms, including an increased flux through the polyol and glucosamine pathways, activation of protein kinase C and NADPH oxidase, and formation of advanced glycation end products [11], [12], [31], [32]. AOPP concentration reflects the oxidation of plasma proteins, especially albumin [13]. Plasma AOPP concentration was shown to be increased in type 2 diabetic patients with diabetic nephropathy [33], [34] and in type 1 diabetic patients with microvascular complications [35] as compared to patients without microvascular complications. Experimental studies in rodents suggest that AOPP enhances oxidative stress in the kidney and is implicated in the pathogenesis of glomerulosclerosis. Intravenous administration of AOPPs in diabetic rats increased AOPP levels in the kidney, promoted renal inflammation, glomerular hypertrophy and overexpression of fibronectin, and led to albuminuria [36]. NADPH oxidase is a major source of ROS production in the kidney of diabetic rats [36], [37] and AOPP was shown to mediate the activation of NADPH oxidase in the rat kidney [36], [38].

The antioxidant enzymes are the first defence line against oxidative stress [39]. It is noteworthy that in our study, AOPP concentration and SOD activity in the plasma were inversely correlated. The protective role of Mn-SOD in the pathophysiology of kidney disease is supported by studies in animal models. Heterozygous *Sod2* deficient mice, as compared to wild-type mice, showed ROS-induced oxidative renal damage, with massive glomerulosclerosis, tubulointerstitial damage and inflammation in the kidney [40]. Pharmacologically-induced normalization of Mn-SOD activity in db/db diabetic mice with Mn-SOD dysfunction decreased oxidative stress and improved renal histological and functional abnormalities, including albuminuria [41]. In this regard, pharmacological inhibition of the renin-angiotensin system was shown to associated with increased antioxidant defences in mouse tissues, including the kidney [42], and with decreased renal protein oxidative damage in diabetic rats [43]. In agreement with these findings, the use of ACE inhibitors in GENEDIAB was associated with ∼48% increase in plasma SOD activity and ∼46% decrease in plasma AOPP concentration. Use of this class of drugs was the major determinant of the variation of both markers in our cohort.

In conclusion, we have observed associations of allelic variations of *SOD2* gene with the incidence and the progression of diabetic nephropathy, with the decline of eGFR, and with plasma AOPP concentration and SOD activity in subjects with type 1 diabetes. Our results are consistent with an implication of oxidative stress in the pathophysiology of diabetic nephropathy and with a major role of antioxidant enzymes as a mechanism of renal protection.

## Supporting Information

Table S1 GENESIS and GENEDIAB pooled studies: Characteristics of participants by diabetic retinopathy status.
(DOC)
