# Supplementary material for: Manganese Superoxide Dismutase (SOD2) Polymorphisms, Plasma Advanced Oxidation Protein Products (AOPP) Concentration and Risk of Kidney Complications in Subjects with Type 1 Diabetes
Source: PLoS One. 2014 May 12;9(5):e96916. doi: 10.1371/journal.pone.0096916 (PMC4018399; doi:10.1371/journal.pone.0096916)
Supplement: Table S1 — GENESIS and GENEDIAB pooled studies: Characteristics of participants by diabetic retinopathy status. (DOC) [file pone.0096916.s001.doc]

***Manuscript PONE-D-13-52233 R1***

**Table S1.** GENESIS and GENEDIAB pooled studies: Characteristics of participants by diabetic retinopathy status

|  | Non-proliferative or Pre-proliferative retinopathy | Proliferative retinopathy | p |
| --- | --- | --- | --- |
| N | 366 | 579 |  |
| Age (years) | 46 ± 11 | 48 ± 12 | 0.04 |
| Sex: M/F (%) | 47 / 53 | 46 / 54 | 0.74 |
| BMI (kg/m^2^) | 24.3 ± 3.4 | 23.8 ± 3.3 | 0.02 |
| Age at diabetes onset (years) | 18 ± 9 | 15 ± 8 | <0.0001 |
| Duration of diabetes (years) | 28 ± 9 | 33 ± 10 | <0.0001 |
| HbA_1c_ (mmol/mol)(%) | 66 ± 17 (8.2 ± 1.6) | 67 ± 17 (8.3 ± 1.6) | 0.59 |
| Systolic blood pressure (mmHg) | 131 ± 16 | 135 ± 20 | 0.002 |
| Diastolic blood pressure (mmHg) | 73 ± 10 | 76 ± 11 | 0.0002 |
| Antihypertensive treatment (%) | 50 | 66 | 0.0008 |
| Plasma creatinine (µmol/l) | 98 ± 73 | 148 ± 140 | <0.0001 |
| eGFR (ml/min) | 82 ± 29 | 63 ± 30 | <0.0001 |
| Urinary albumin excretion (mg/l)* | 14 (53) | 49 (556) | <0.0001 |
| Diabetic nephropathy stages (%) | 51 / 26 / 13 / 10 | 29 / 20 / 21 / 30 | <0.0001 |
| ACE Inhibitor treatment (%) | 47 | 55 | 0.03 |
| Tobacco smoking^‡^ | 36.7 | 39.9 | 0.32 |
| Total Cholesterol (mmol/l)**^§^** | 5.72 ± 1.49 | 5.67 ± 1.44 | 0.71 |
| Triglycerides (mmol/l)***^§^** | 1.08 (0.73) | 1.00 (0.81) | 0.84 |
| Hypolipemic Treatment (%) | 7.7 | 8.9 | 0.50 |
| Myocardial infarction (%) | 4.2 | 9.2 | 0.01 |
| Macrovascular complications (%) | 11.5 | 22.6 | <0.0001 |

Results expressed as means ± SD or *median and interquartile range. Statistics of quantitative parameters are ANOVA performed with log-transformed data or *Wilcoxon (rank sums) test. p<0.05 is significant. Diabetic nephropathy stages: Absent, Incipient, Established, Advanced. ^§^Total cholesterol (n=438) and triglycerides (n=127) concentrations available only for GENEDIAB participants. Macrovascular complications include all cases of myocardial infarction, stroke and/or peripheral artery disease. **^‡^**Current or past history of tobacco smoking.
